# Mental Representation and Mental Practice: Experimental Investigation on the Functional Links between Motor Memory and Motor Imagery

**DOI:** 10.1371/journal.pone.0095175

**Published:** 2014-04-17

**Authors:** Cornelia Frank, William M. Land, Carmen Popp, Thomas Schack

**Affiliations:** 1 Neurocognition and Action - Biomechanics Research Group, Faculty of Psychology and Sports Science, Bielefeld University, Bielefeld, Germany; 2 Cognitive Interaction Technology - Center of Excellence (CITEC), Bielefeld University, Bielefeld, Germany; 3 Research Institute for Cognition and Robotics (CoR-Lab), Bielefeld University, Bielefeld, Germany; 4 Department of Kinesiology, Health, & Nutrition, University of Texas at San Antonio, San Antonio, Texas, United States of America; University of Udine, Italy

## Abstract

Recent research on mental representation of complex action has revealed distinct differences in the structure of representational frameworks between experts and novices. More recently, research on the development of mental representation structure has elicited functional changes in novices' representations as a result of practice. However, research investigating if and how mental practice adds to this adaptation process is lacking. In the present study, we examined the influence of mental practice (i.e., motor imagery rehearsal) on both putting performance and the development of one's representation of the golf putt during early skill acquisition. Novice golfers (*N* = 52) practiced the task of golf putting under one of four different practice conditions: mental, physical, mental-physical combined, and no practice. Participants were tested prior to and after a practice phase, as well as after a three day retention interval. Mental representation structures of the putt were measured, using the structural dimensional analysis of mental representation. This method provides psychometric data on the distances and groupings of basic action concepts in long-term memory. Additionally, putting accuracy and putting consistency were measured using two-dimensional error scores of each putt. Findings revealed significant performance improvements over the course of practice together with functional adaptations in mental representation structure. Interestingly, after three days of practice, the mental representations of participants who incorporated mental practice into their practice regime displayed representation structures that were more similar to a functional structure than did participants who did not incorporate mental practice. The findings of the present study suggest that mental practice promotes the cognitive adaptation process during motor learning, leading to more elaborate representations than physical practice only.

## Introduction

According to skill acquisition theories, cognitive mechanisms governing skill execution develop over the course of learning [1]–[5]. To this extent, skill acquisition is known to be accompanied by both overt changes (i.e., performance improvements) and covert changes (i.e., cognitive improvements) over time. Of particular interest for skill acquisition is the role that mental representations play in the learning and control of actions. Individuals of different skill levels have been suggested to differ not only in their overt performance [6], but also in their underlying skill representations in long-term memory [7]–[11]. Consequently, an individual's mental representation of a motor skill is thought to change on his/her way to expertise, namely in the direction of an elaborate, well-developed representation [12].

Knowledge-based mental representation structures in long-term memory have been measured using a variety of different methods [13]. One approach, which specifically takes into account the cognitive level of motor actions, is the cognitive action architecture approach (CAA-A) [14]–[16]. According to this approach, motor learning can be characterized as the modification and adaptation of representational frameworks of complex actions in memory. Representational frameworks are comprised of basic action concepts (BACs; i.e., cognitive chunks of movement postures and their sensory consequences within the realization of an action goal), which reflect the building blocks of an action in long-term memory.

Early research on representational frameworks of complex action has elicited distinct differences in the mental representation between experts and novices. Schack and Mechsner [15], for example, investigated representational frameworks of the tennis serve in expert and non-expert tennis players using structural dimensional analysis of mental representation (SDA-M) [14], [17]. Findings revealed distinct differences between the mental representation of expert and novice tennis players such that experts' structures were more elaborate than novices' structures. More specifically, whereas the mental representations of experts were organized hierarchically and structured in a functional way (i.e., BACs being grouped according to the functional and biomechanical demands of the tennis serve), the mental representations of novices were not. Moreover, novices' mental representations varied greatly in their structure, while those of experts were more similar. From this, the authors concluded that such elaborate skill representations in long-term memory play a salient role in skilled action. Up to now, distinct differences in representational frameworks of complex action have been demonstrated across a variety of sports, such as dance [18], volleyball [19], and windsurfing [20]. Furthermore, the results have been shown to generalize to developmental aspects of manual action [21], and to special populations [22].

More recently, Frank, Land, and Schack [23] examined if and how representational frameworks of complex action change over the course of practice in early skill acquisition. Specifically, a group of novices practiced a putting task over the course of three days, whereas a control group did not putt at all. Mental representation structures were recorded prior to and after practice as well as after a three-day retention interval. Results indicated that neither of the groups' mental representations revealed any meaningful structure of the putt prior to practice. However, along with performance improvements, changes in the mental representation structure were evident for the practice group. Specifically, after substantial putting practice, the mental representation of the practice group revealed a structure that reflected key parts of the movement phase pertaining to the functional and biomechanical demands of the task. For the control group, however, no changes in mental representation of the putt were evident from pre-, to post- and to retention-test. From this, it was concluded that the acquisition of motor skills is associated with functional adaptations of the representational frameworks in long-term memory. In addition to the research showing the changes in mental representation over the course of skill acquisition, more recently, Land, Frank, & Schack [24] demonstrated that the type of instructions given to novices during learning (here: internal vs. external focus) can influence the rate of representation development. Results indicated that learners instructed to adopt an external focus of attention performed with greater putting accuracy and consistency, while also revealing a greater degree of development in their mental representation of the putting task.

Interestingly, while instructional type has been shown to influence the development of mental representations during skill acquisition, research to date has yet to consider the influence that mental practice can have on this process. As an important means to promote motor skill acquisition, mental practice has received a great deal of attention in the last 50 years within cognitive sport psychology. Mental practice in the sense of motor imagery rehearsal refers to the act of repeatedly simulating (i.e., imagining) a motor action in one's mind without actually executing it at the same time [25]–[28]. Unlike perception, imagery can be understood as the creation or re-creation of real-world experiences in the absence of the actual sensory stimuli [29]–[31]. Accordingly, in contrast to actual or physical practice, which implies overtly rehearsing a motor action, mental practice in the sense of motor imagery rehearsal refers to the covert rehearsal of a motor action by way of imagery.

Up to now, mental practice has proven to be an effective tool, both to improve performance and to promote learning [32]–[36]. Meta-analyses studying the effectiveness of mental practice have reported small to moderate effect sizes (i.e., *d* = .48 to *d* = .68), suggesting that mental practice, although not as effective as physical practice, significantly influences performance compared to no practice. While, to date, no meta-analysis exists that has thoroughly examined the effectiveness of a combination of physical and mental practice, findings from various studies support the superiority of such a combined type of practice on performance [37]–[39]. From this and other research, mental practice can be considered as an effective means to improve performance and to promote learning. Specifically, comparing the effectiveness of each practice type (i.e., combined practice (CP) – physical practice (PP) – mental practice (MP) – no practice (NP)), combined practice has been shown to be most effective, followed by physical practice, while mental practice is less effective than its physical counterpart, but more effective than no practice (i.e., CP>PP>MP>NP).

Researchers have suggested a variety of possible explanations for the underlying mechanisms of mental practice [31], [35]. Two early theories offer two distinct perspectives, one focusing on more peripheral processes (i.e., psychoneuromuscular theory) [40], and one focusing on more central mechanisms (i.e., symbolic learning theory) [41]. The psychoneuromuscular theory [40] is centered around the activation of muscles during imagery. According to this theory, mental practice is thought to facilitate the performance and the learning of a movement such that it causes a similar activation pattern of muscles as during movement execution, which in turn aids subsequent movement execution. In contrast to this more peripheral motor explanation, the symbolic learning theory [41], representing a cognitive explanation, proposes that the sequence of a movement is coded through symbols. Accordingly, mental practice is thought to facilitate performing a movement sequence through the repetition of symbolic components of the movement sequence resulting in a better symbolic representation.

More recently, the increasing interest in and findings from neurophysiological research have led to an explanation for the effects of mental practice which is known as the principle of functional equivalence [27], [28], [42], [43]. This principle focuses on central mechanisms as well, and as such proposes that the simulation of a movement (i.e., motor imagery) and the execution of a movement are functionally equivalent. Thus, as stated by the functional equivalence principle, mental practice to some extent involves the same underlying structures and covert processes as physical practice. Specifically, during motor imagery, the mental representation of a motor action is activated in order to enable the imager to imagine the movement, and it is stabilized as a result of repeatedly imagining the movement. In this sense, mental practice is thought to help improve performance and learning in a functionally equivalent way as physical practice does. Up to now, findings from neurophysiological research mainly support the functional equivalence between the simulation and the execution of an action [27], [44]–[46]. Moreover, neurophysiological studies have shown that both mental and physical practice lead to significant changes in neural networks during skill acquisition [47]–[51]. However, although neurophysiological studies elicit changes in brain activation following mental practice, it is not clear, what these changes stand for on a cognitive representational level. Such changes in neurophysiological variables point to the idea that functional changes on a cognitive level (i.e., concept formation in one's mental representation) may take place during mental practice.

Taken together, while the acquisition of a complex motor skill by way of physical practice has been shown to be accompanied by the formation of representation structures in long-term memory, it is currently unclear how mental practice affects this representation formation process. Analogous to changes in brain activation on a neural level, mental practice may lead to functional adaptations in mental representation on a cognitive level. That is, we expect mental practice to add to the development of representation structures. Moreover, examining the effect of mental practice on both the overt level of performance and the covert level of mental representations in novices might help to gain more detailed understanding of the covert processes that do or do not lead to performance improvements and learning in early skill acquisition. To date, research examining how mental practice affects both overt motor performance and covert mental representation is lacking. Hence, with the present study, recreating the typical four groups mental practice design [34], [52], we aim at bridging this gap by examining the effects of mental practice on both the performance level and the mental representation level. In short, we examined how physical practice, mental practice, and a combination of both affect the performance and the development of one's mental representation of a golf putting task. Based on previous findings, it was predicted that putting performance would change according to type of practice such that combined practice would be superior to physical practice, which in turn would be superior to mental practice (i.e., CP>PP>MP>NP). Furthermore, it was predicted that, along with performance improvements, changes to the underlying mental representation would be evident as a consequence of skill acquisition. Specifically, it was predicted that novices' unstructured mental representation would turn into a more structured representation with practice. More importantly however, we were interested in what impact mental practice would have on mental representation development, and whether this related to performance.

## Methods

### Participants

Fifty-two students participated in the present study. All participants were novice golfers with no prior experience in golf. Each participant was randomly assigned to one of four groups: mental practice group (*n* = 13, mean age = 23.15, *SD* = 2.28, 8 female), physical practice group (*n* = 13, mean age = 24.54, *SD* = 3.64, 9 female), mental-physical combined practice group (*n* = 13, mean age = 23.69, *SD* = 2.93, 9 female) and no practice group (*n* = 13, mean age = 27.31, *SD* = 5.53, 8 female). The experimental procedure and written consent form for this study were approved by the ethics committee at Bielefeld University, and adhered to the ethical standards of the sixth revision of the Declaration of Helsinki. All participants gave their informed written consent to participate in the study.

### Tasks and Measures

#### Performance

A standard putter and a standard golf ball were used in the present study. Golf putts were performed on an artificial indoor putting green (size: 4×7 m). Participants performed putts to a target three meters away from the starting point. Specifically, participants were instructed to putt a golf ball as accurately as possible to the target, on which the ball was supposed to stop. The target was marked by a circle 10.8 cm (4.25 in) in diameter in accordance with the size of a regular golf hole. The outcome of each golf putt was recorded by capturing the final ball position after each putt with a motion capture system. Specifically, 6 T10 CCD cameras captured and tracked the golf ball rolling and stopping, with a spatial resolution of approximately 0.25 mm and a temporal resolution of 200 Hz.

#### Mental representation structure

In order to assess mental representation structure, we employed structural dimensional analysis of mental representation (SDA-M). This method provides psychometric data on the structure and dimension of mental representations of complex movements in long-term memory. More specifically, the SDA-M proceeds in four steps: (1) a split procedure delivering a distance scaling between the BACs of a suitably predetermined set, (2) a hierarchical cluster analysis used to outline the structure of the given set of BACs, (3) a factor analysis revealing the dimensions in this structured set of BACs, and (4) an analysis of invariance within- and between-groups in order to compare different cluster solutions [17]. More specifically, in order to determine distances between BACs in memory, mental representation structure was assessed by way of a splitting task, first step of the SDA-M described above. The splitting task operates as follows: one BAC of the putt is permanently displayed on a computer screen (i.e., the anchor concept), while the rest of the concepts are presented one after another in randomized order. Participants are instructed to indicate whether a given BAC is related to the anchor concept or not during movement execution. As soon as a list of BACs is finished, another BAC takes the anchor position and the procedure continues. The splitting task is completed after each BAC has been compared to the remaining BACs (n-1).

In order to examine the underlying representation structure of the putt, the BACs of the movement have been adopted from Frank et al. [23]. Accordingly, the following 16 BACs for the putt were used in the present study: (1) shoulders parallel to target line, (2) align club face square to target line, (3) grip check, (4) look to the hole, (5) rotate shoulders away from the ball, (6) keep arms-shoulder triangle, (7) smooth transition, (8) rotate shoulders towards the ball, (9) accelerate club, (10) impact with the ball, (11) club face square to target line at impact, (12) follow-through, (13) rotate shoulders through the ball, (14) decelerate club, (15) direct clubhead to planned position, (16) look to the outcome. Each of these 16 BACs of the putt can be designated to one movement phase: preparation (BAC 1–4), backswing (BAC 5–7), forward swing (BAC 8–9), impact (10–13) and attenuation (BAC 14–16).

#### Imagery ability

Visual and kinesthetic imagery ability was measured using the revised version of the Movement Imagery Questionnaire (MIQ-R) [53]. Accordingly, participants were asked to perform, imagine and finally rate their imagery experience of a series of movements. More specifically, after having performed a given movement, participants were instructed to either “see” or “feel” the movement without actually performing it. Next, they were asked to rate the ease or difficulty of imagining the movement on a 7-point Likert scale. This procedure was repeated for 4 different movements, and for both visual and kinesthetic imagery, resulting in 8 items.

#### Manipulation check

For the two groups involving mental practice in their practice regime, as suggested by Goginsky and Collins [54], a post-experimental questionnaire was administered following each practice session in order to investigate whether participants performed the imagery as instructed. Specifically, participants of the mental practice groups were asked to describe the content of their imagery in detail. In addition, they had to indicate on 7-point Likert scales (1 =  *very difficult*, 7 =  *very easy*), how easy it was for them to follow the instructions in general, as well as how easy it was to “see” and how easy it was to “feel” the movement in particular. Also, participants were asked how often they used an external perspective and how often they used an internal perspective (7-point Likert scales; 1 =  *never*, 7 =  *always*) during their imagery. Furthermore, they were asked whether they had experienced any problems, and whether they had any previous experience with imagery.

### Procedure

The present study consisted of a pre-test, an acquisition phase on three consecutive days, followed by a post-test and a retention test 72 hours later (see Table 1).

**Table 1:** Design of the study including three test days and an acquisition phase.

	Pre-test	Acquisition			Post-test	Retention-test
	Day 1	Day 2	Day 3	Day 4	Day 5	Day 8
**Combined practice group** (*n* = 13)	SDA-M		-		SDA-M	SDA-M
	Putting task	Putting practice (executed and imagined putts)			Putting task	Putting task
**Physical practice group** (*n* = 13)	SDA-M		-		SDA-M	SDA-M
	Putting task	Putting practice (executed putts only)			Putting task	Putting task
**Mental practice group** (*n* = 13)	SDA-M		-		SDA-M	SDA-M
	Putting task	Putting practice (imagined putts only)			Putting task	Putting task
**Control group** (*n* = 13)	SDA-M		-		SDA-M	SDA-M
	Putting task	No putting practice (reading)			Putting task	Putting task

*Note*: SDA-M: structural dimensional analysis of mental representation; putting task on test days: 3×20 putts; putting practice during acquisition phase: 3×20 (imagined or/and executed) putts per day (practice groups) or 20 min of reading per day (control group).

#### Pre-test

On the first day, each participant signed informed consent forms. In order to become familiar with the movement, each participant watched a video of a skilled golfer performing the putting task. An introduction to the splitting task by the experimenter followed (for details on the SDA-M, see Tasks and Measures section). Before completing the splitting task, each participant was presented a randomized list of the 16 BACs of the putt. In order to ensure comprehension of the concepts, the experimenter explained the meaning of each of the 16 BACs to the participant. Next, the participants read the instructions on how to complete the splitting task. Specifically, participants were asked to decide whether the presented BACs are related to one another or not during movement execution. Following, the participants completed the splitting task. This procedure served to determine their starting mental representation structure of the putt. In order to assess their starting performance level, each participant then performed three blocks of 20 putts each. They were instructed to putt a golf ball as accurately as possible to the target, on which the ball was supposed to stop. As a measure of imagery ability, each participant completed the MIQ-R.

#### Practice phase

The next three days, participants of each practice group performed three blocks of twenty putts each (either physically or mentally or a combination of both), while participants of the control group did not practice the putt at all.

##### **Physical practice (PP) group**

Physical practice consisted of three blocks of 20 actual putts on each day of the practice phase. Specifically, participants were instructed to putt as accurately as possible to the target, on which the ball was supposed to stop. No additional information (e.g., technical feedback) was given. The visible outcome of the putt (i.e., knowledge of result) was the only feedback available for the participants.

##### **Mental practice (MP) group**

Mental practice on each practice day was comprised of specific motor imagery (i.e., putting imagery). Participants in this group did not physically execute the putt during practice. The motor imagery consisted of three blocks of 20 imagined putts each with a short break between the blocks. More specifically, each participant was asked to take the starting position as if they were going to actually putt. That is, participants stood upright on the green with the putter in their hands and their eyes closed. Next, the imagery script was read out loud to each participant, both at the beginning and before each block. Predefined by the script, participants were asked to imagine both the putting movement as well as the ball rolling toward the target and stopping on the target (for more details, see imagery scripts [scripts are available from the corresponding author upon request]). In order to control for as many aspects during imagery as possible and to optimize the efficacy of the imagery intervention, participants were further told to imagine from an internal perspective (i.e., imagery perspective), to incorporate all the senses in their imagery (i.e., imagery modality), and to try and imagine as clear and as vivid as possible (i.e., imagery vividness) [55]. After the script was read, participants imagined repeatedly the putting movement on their own. In order to enable the experimenter to control for the intended number of putts, participants were asked to indicate when having finished one putt in their imagery by slightly raising their index finger. Following imagery, participants of the mental practice group filled out a post-experimental questionnaire.

##### **Combined practice (mental and physical practice; CP) group**

The combined practice consisted of three blocks of twenty putts on each day of the practice phase, with each block consisting of 10 imagined followed by 10 actual putts (for specific instructions for each of the two types of practice, see both the physical practice group and mental practice group descriptions).

##### **No practice (control; NP) group**

The control group neither imagined nor executed the putting movement during the practice phase. Instead, participants in the control group were asked to read about golf in general in “Dream on: one hack golfer's challenge to break par in a year” [56]. The reading lasted for twenty minutes each day, which is approximately the time needed to imagine three blocks of 20 putts.

#### Post- and retention-test

In order to determine their final mental representation structures of the putting movement, all participants completed the splitting task again, one day after acquisition phase as well as after a retention interval of three days. In addition, each participant performed three blocks of 20 putts once more to assess their final outcome performance for post- and retention-test.

### Data Analysis

#### Mental representation structure

The structure of mental representations was assessed by way of cluster analysis resulting in mean group dendrograms [17]. For all cluster analyses conducted, an alpha-level of *α* = .05 was chosen, resulting in a critical value *d*_crit_ = 3.41. BACs linked above this critical value were considered irrelevant. That is, links between concepts above this value were considered not related, while concepts linked below this value were considered related and thus resulted in a cluster. In order to compare differences between cluster solutions, analyses of invariance were conducted [17], [57], [58]. Accordingly, cluster solutions are variant (i.e., differ), for λ<0.68, while cluster solutions are invariant (i.e., do not differ) for λ≥0.68. Moreover, the Adjusted Rand Index (ARI) [59], [60] was used to further investigate the degree of similarity between mean group dendrograms and a reference dendrogram reflecting the different movement phases. The Adjusted Rand Index is an index of similarity, ranging on a scale from −1 to 1. As the value “−1” denotes that cluster solutions are different and the value “1” denotes that two cluster solutions are the same, indices between “−1” and “1” mark the degree of similarity between two cluster solutions.

#### Performance

Putting performance was measured by two outcome variables (i.e., accuracy and consistency) for each time of measurement. Specifically, accuracy and consistency were calculated using two-dimensional error scores based on the x and y coordinates of each putt with the center of the target being the origin of the axes [61]. Accuracy was measured by mean radial error (MRE), defined as a subject's average distance each putt came to the center of the target in centimeters. Consistency was measured by bivariate variable error (BVE), analogous to variable error in one-dimensional analyses, and defined as the square root of a subject's k shots' mean squared distance from their centroids in centimeters. A subject's centroid is a positionally typical shot whose coordinates are given by the average x and average y value of a subject's shots in centimeters. Learning over time was analyzed by way of two separate one-way MANCOVAs on both the post-test scores and the retention-test scores of the two dependent variables MRE and BVE. Specifically, a one-way MANCOVA on post-test scores with group as a between-subjects factor and pre-test scores as a covariate was conducted in order to examine whether the groups differed in their performance after acquisition phase as a result of practice condition, thereby controlling for potential differences in their pre-test performance. Regarding retention, a one-way MANCOVA on retention-test scores with group as a between-subjects factor and pre-test scores as a covariate was performed in order to examine whether the groups differed in their level of performance after a three day period of no practice, while controlling for the level of performance at baseline. Next, separate one-way ANCOVAs were conducted for each of the dependent variables. As directional effects had been specified a priori (CP>PP>MP>NP), one-tailed pairwise comparisons based on estimated marginal means served as tests of significance. A Holm-Bonferroni correction was employed in order to account for the inflation of type I errors [62]. Cohen's *d* was used as an estimate of effect size [63].

#### Imagery ability

In order to compare imagery ability between groups, three separate one-way ANOVAs on overall imagery ability (i.e., both scales together) as well as on visual and kinesthetic imagery ability were conducted.

## Results

### Imagery Ability

Overall, participants reported acceptable visual imagery ability (*M* = 21.46, *SD* = 3.84.; 5.37 per item) as well as acceptable kinesthetic imagery ability (*M* = 19.77, *SD* = 4.47.; 4.94 per item). Specifically, on average participants scored approximately 5 on both scales (i.e., *somewhat easy to see/feel*), which is considered as sufficient imagery ability for subsequent mental practice sessions [64], [65]. In addition, one-way ANOVAs on imagery ability revealed no main effect of group, neither for overall imagery ability, *F*(3,48) = .273, *p* = .845, *η_p_* ^2^ = .017, nor for visual imagery ability, *F*(3,48) = .170, *p* = .916, *η_p_* ^2^ = .011, or kinesthetic imagery ability, *F*(3,48) = .198, *p* = .897, *η_p_* ^2^ = .012, indicating that imagery ability was similar for each of the four groups.

### Manipulation Check

In order to ensure that participants of the mental and mental-physical combined practice group had performed the imagery as instructed, participants' manipulation check responses were analyzed. None of the participants had prior imagery experience. In addition, none of the participants reported any problems during imagery sessions. Relating to the content of imagery, each participant mentioned the putting movement as well as the ball rolling in their descriptions of imagery content. Furthermore, for imagery perspective, mean scores during practice phase were 6.40, *very often* (*SD* = 0.53) for internal perspective and 1.80, *almost never* (*SD* = 0.85) for external perspective, indicating that participants of the mental practice and the mental-physical combined practice group had adopted an internal perspective during imagery. For ease of visual and kinesthetic imagery, participants scored an average of 4.37, *neither easy nor difficult* (*SD* = 1.40) for visual imagery and 4.67, *somewhat easy to feel* (*SD* = 1.49) for kinesthetic imagery, meaning that they had been able to “see” and to “feel” the movement while imagining. For instructions in general, mean scores were 4.73, *somewhat easy* (*SD* = 1.29), indicating that participants had been able to follow the instructions during imagery. Thus, participants had been able to perform the imagery as instructed, which was considered a prerequisite for subsequent data analyses.

### Mental Representation Structure

**Figure 1:**
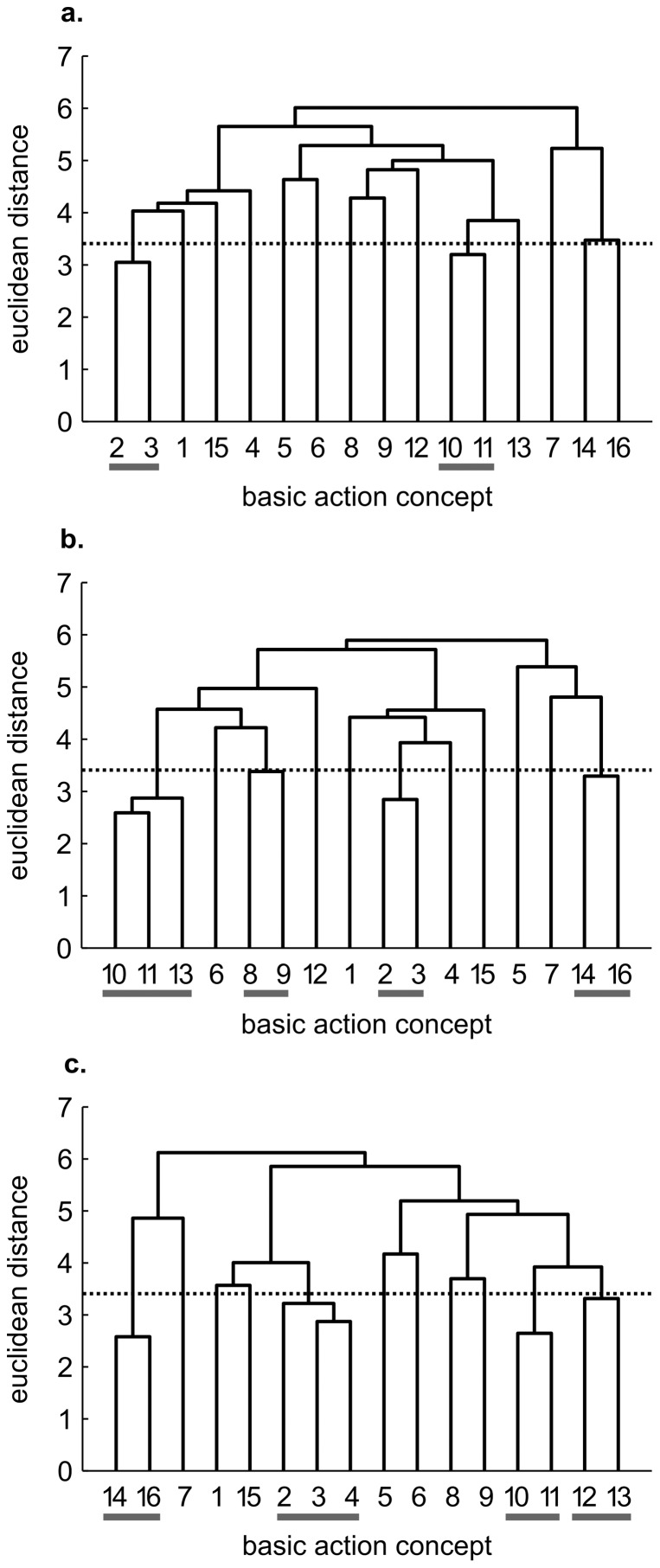
Mean group dendrograms of the mental practice group (*n* = 13) for the golf putt. The dendrograms refer to (a) pre-test, (b) post-test and (c) retention-test. The numbers on the x-axis relate to the BAC number, the numbers on the y-axis display Euclidean distances. The lower the link between related BACs, the lower is the Euclidean distance. The horizontal dotted line marks *d_crit_* for a given *α*-level (*d_crit_ = 3.41*; *α* = .05): links between BACs above this line are considered not related; horizontal grey lines on the bottom mark clusters. BACs: (1) shoulders parallel to target line, (2) align club face square to target line, (3) grip check, (4) look to the hole, (5) rotate shoulders away from the ball, (6) keep arms-shoulder triangle, (7) smooth transition, (8) rotate shoulders towards the ball, (9) accelerate club, (10) impact with the ball, (11) club face square to target line at impact, (12) follow-through, (13) rotate shoulders through the ball, (14) decelerate club, (15) direct clubhead to planned position, and (16) look to the outcome.

**Figure 2:**
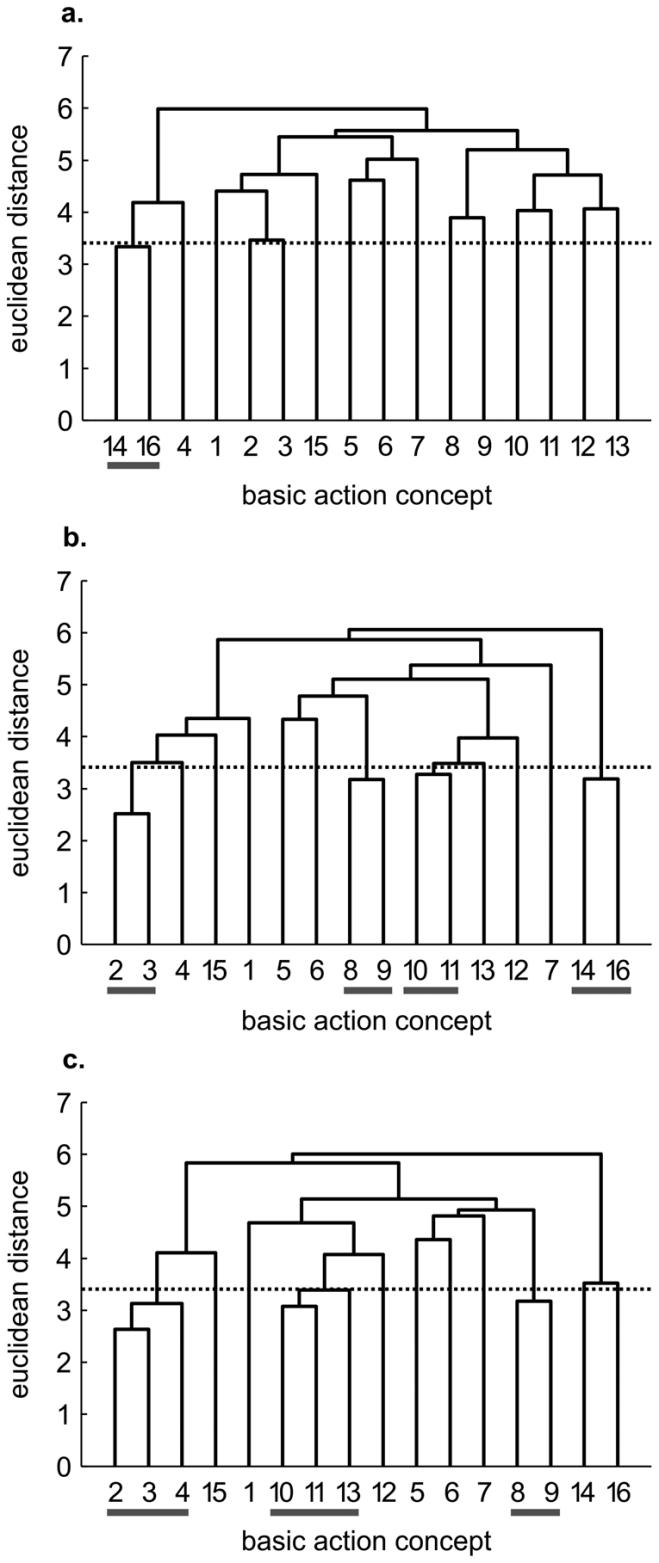
Mean group dendrograms of the combined practice group (*n* = 13) for the golf putt. The dendrograms refer to (a) pre-test, (b) post-test and (c) retention-test (*α* = 0.05; *d_krit_* = 3.41).

**Figure 3:**
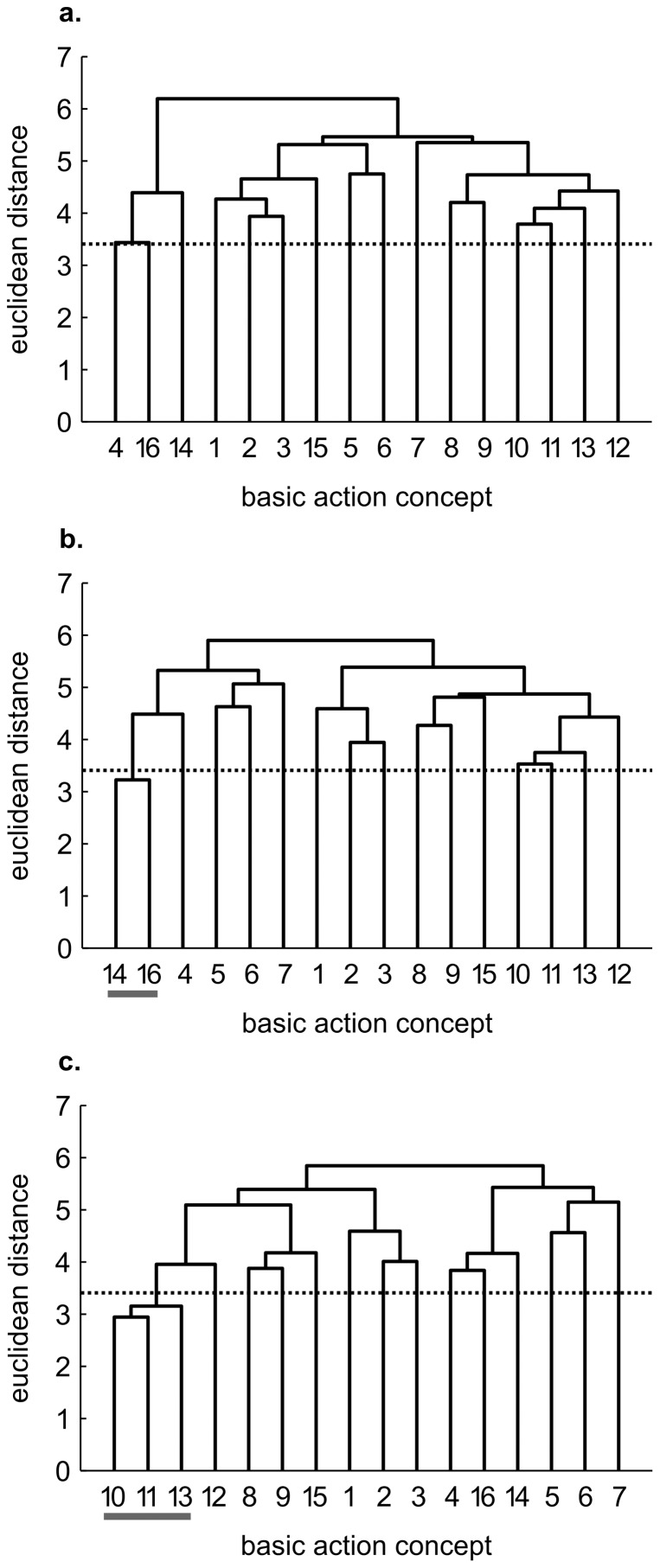
Mean group dendrograms of the physical practice group (*n* = 13) for the golf putt. The dendrograms refer to (a) pre-test, (b) post-test and (c) retention-test (*α* = 0.05; *d_krit_* = 3.41).

While cluster analysis revealed little to no clustering in the mean group dendrograms of each group for pre-test, each practice group's dendrograms revealed changes over time (see Figures 1–3).

#### Mental practice group

While no distinct structure existed for the mental practice group at pre-test, a more elaborate mental representation structure was evident after acquisition phase (see Figure 1). More specifically, four functional clusters were observed in the mental practice group's mean dendrogram at post-test, pertaining to three phases of the putt: preparation (i.e., BAC 2, 3), forward swing and impact (i.e., BAC 8, 9 as well as BAC 10, 11, 13), and attenuation (i.e., BAC 14, 16). The same was true for retention-test with some minor differences for impact phase (i.e., two separate clusters: BAC 10, 11 as well as 12, 13). Thus, for the mental practice group, an increase in the number of functional clusters was apparent in their mental representation structure over the course of the study. Statistical analyses of invariance confirmed the above presented descriptive results, revealing significant differences in representation structure between pre- and post-test, pre- and retention-test, as well as between post- and retention-test (λ≤0.68). What is more, increasing adjusted rand indices from pre-test (ARI = 0.17) to post-test (ARI = 0.44) and to retention-test (ARI = 0.44) indicated that, over the course of mental practice, the mean dendrograms of the mental practice group became more similar to the reference dendrogram (for an overview of ARIs, see Table 2). Hence, the changes in representation structure of the mental practice group are functional, and reflect a development towards an optimal structure.

**Table 2:** Degrees of change in adjusted rand indices over the course of the study.

	Degree of change in adjusted rand indices		
	Pre-to post-test	Pre-to-retention-test	Post-to-retention-test
**Combined practice**	0.22	0.41	0.19
**Mental practice**	0.27	0.27	0.00
**Physical practice**	0.09	0.25	0.15
**No practice**	0.08	0.17	0.09

*Note*: The adjusted rand index serves as an index of similarity on a scale from −1 to 1. On this scale, the value “−1” indicates that two cluster solutions (here: mean group dendrograms and the reference) are different and the value “1” indicates that two cluster solutions are the same. Indices between these extremes rank similarity between two cluster solutions.

#### Combined practice group

Similar to the mental practice group, the mental representation structure of the combined practice group was more elaborate after acquisition phase (see Figure 2). Again, four functional clusters were evident in the combined practice group's mean dendrogram at post-test, pertaining to preparation (i.e., BAC 2, 3), forward swing and impact phase (i.e., BAC 8, 9 as well as BAC 10, 11), and attenuation (i.e., BAC 14, 16). For retention-test, the mean group dendrogram revealed basically the same structure with some minor differences in the preparation (i.e., comprised of one additional concept: BAC 2, 3, 4) and the forward swing and impact phase (i.e., BAC 8, 9 and BAC 10, 11, 13). Hence, for the combined practice group, the number of functional clusters increased as well over the course of the study. Statistical analyses of invariance indicated significant differences in representation structure between pre- and post-test, pre- and retention-test, as well as between post- and retention-test (λ≤0.68). When being compared to the reference structure, increasing adjusted rand indices from pre-test (ARI = 0.09) to post-test (ARI = 0.31) and retention-test (ARI = 0.50) were evident, confirming that the mental representation structure of the combined practice group developed towards the reference structure over the course of the study.

#### Physical practice group

In contrast to the mental and the mental-physical combined practice groups, only minor changes in the mental representation structure of the putt were evident for the physical practice group (see Figure 3). Specifically, while the mean group dendrogram of the practice group revealed no cluster at pre-test, the dendrograms revealed one cluster for post-test (i.e., attenuation: BAC 14, 16). For retention-test, one meaningful cluster pertaining to the impact phase (i.e., BAC 10, 11, 13) was evident. Statistical analyses of invariance revealed significant differences between pre- and post-test, pre- and retention-test, as well as between post- and retention-test (λ≤0.68). Interestingly, the practice group's structure revealed only small changes toward the reference structure, with ARI increasing from pre-test (ARI = 0.00) to post-test (ARI = 0.09), and to retention-test (ARI = 0.24).

#### Control group

**Figure 4:**
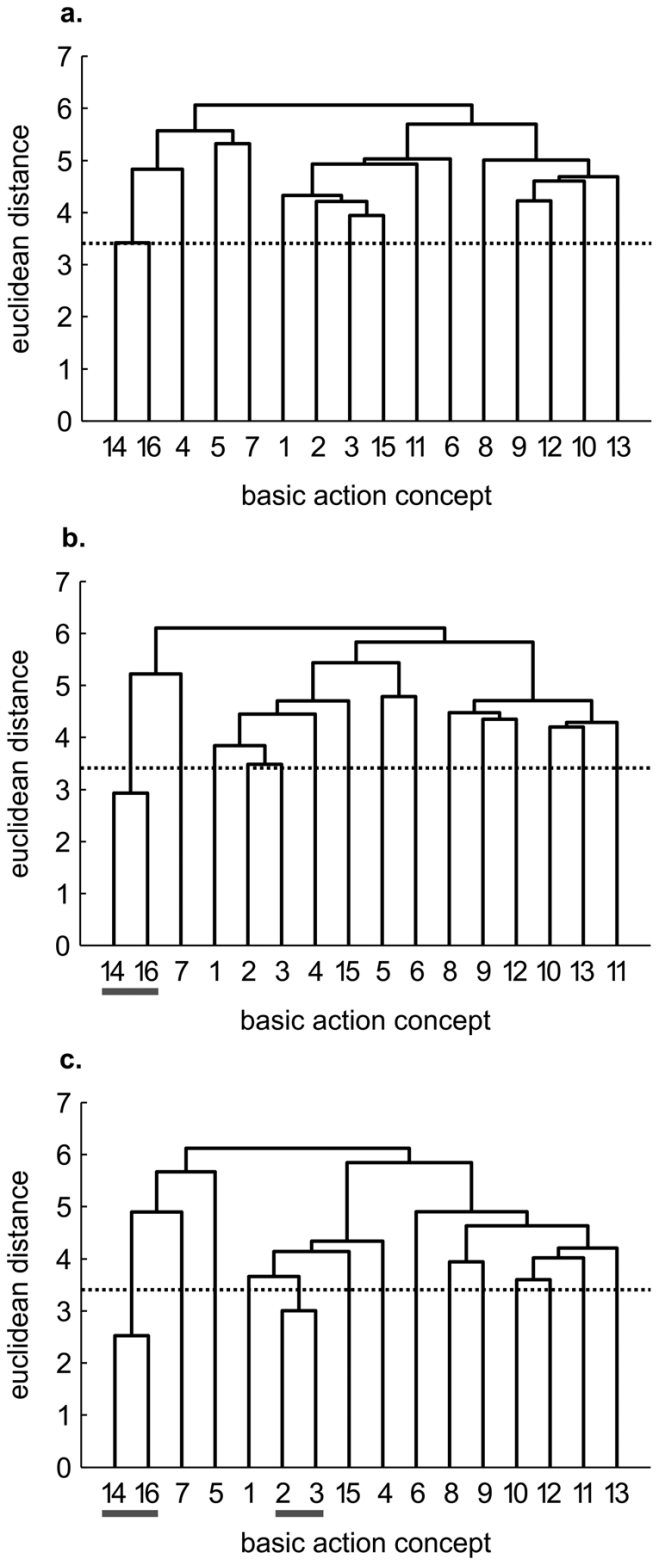
Mean group dendrograms of the control group (*n* = 13) for the golf putt. The dendrograms refer to (a) pre-test, (b) post-test and (c) retention-test (*α* = 0.05; *d_krit_* = 3.41).

For the control group, changes in mental representation structure were small (Figure 4). Specifically, while there were no clusters evident at pre-test, the control group's dendrogram revealed one cluster pertaining to aspects of attenuation of the putting stroke (i.e., BAC 14, 16) at post-test. After the retention interval, the mean dendrogram additionally revealed a second cluster reflecting parts of the preparation (i.e., BAC 2, 3). Statistical analyses of invariance indicated significant differences in representation structure between pre- and post-test, between pre- and retention-test, as well as between post- and retention-test (λ≤0.68). Furthermore, in comparison to the reference structure, the control group's structure showed only a slight trend towards that structure over time, with ARI increasing from pre-test (ARI = 0.00), to post-test (ARI = 0.08), and to retention-test (ARI = 0.17).

Thus, each group's mental representation changed over the course of practice. Moreover, each group's structure developed to some extent in direction of the reference structure. More importantly, whereas the control and the physical practice groups' mental representations elicited only minor changes over the course of the study and showed only a small development towards the reference structure, the representation structures of the mental and the mental-physical combined practice group changed more, and approached more so an optimal representation.

### Outcome Performance

**Figure 5:**
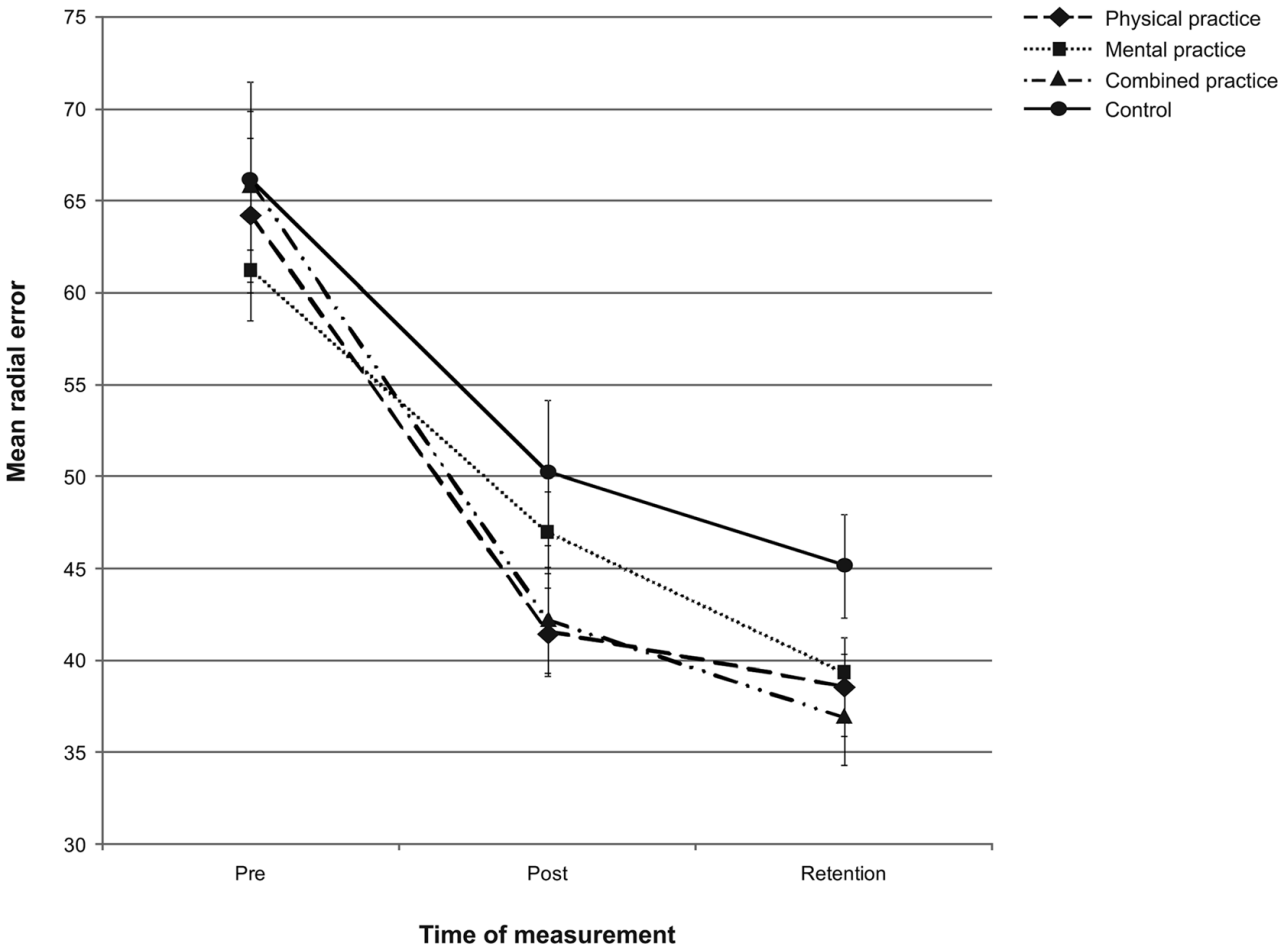
Putting accuracy. Mean radial error (i.e., accuracy) in cm from pre-test to post- and retention-test. The different lines relate to the different groups. Error bars represent standard errors.

**Figure 6:**
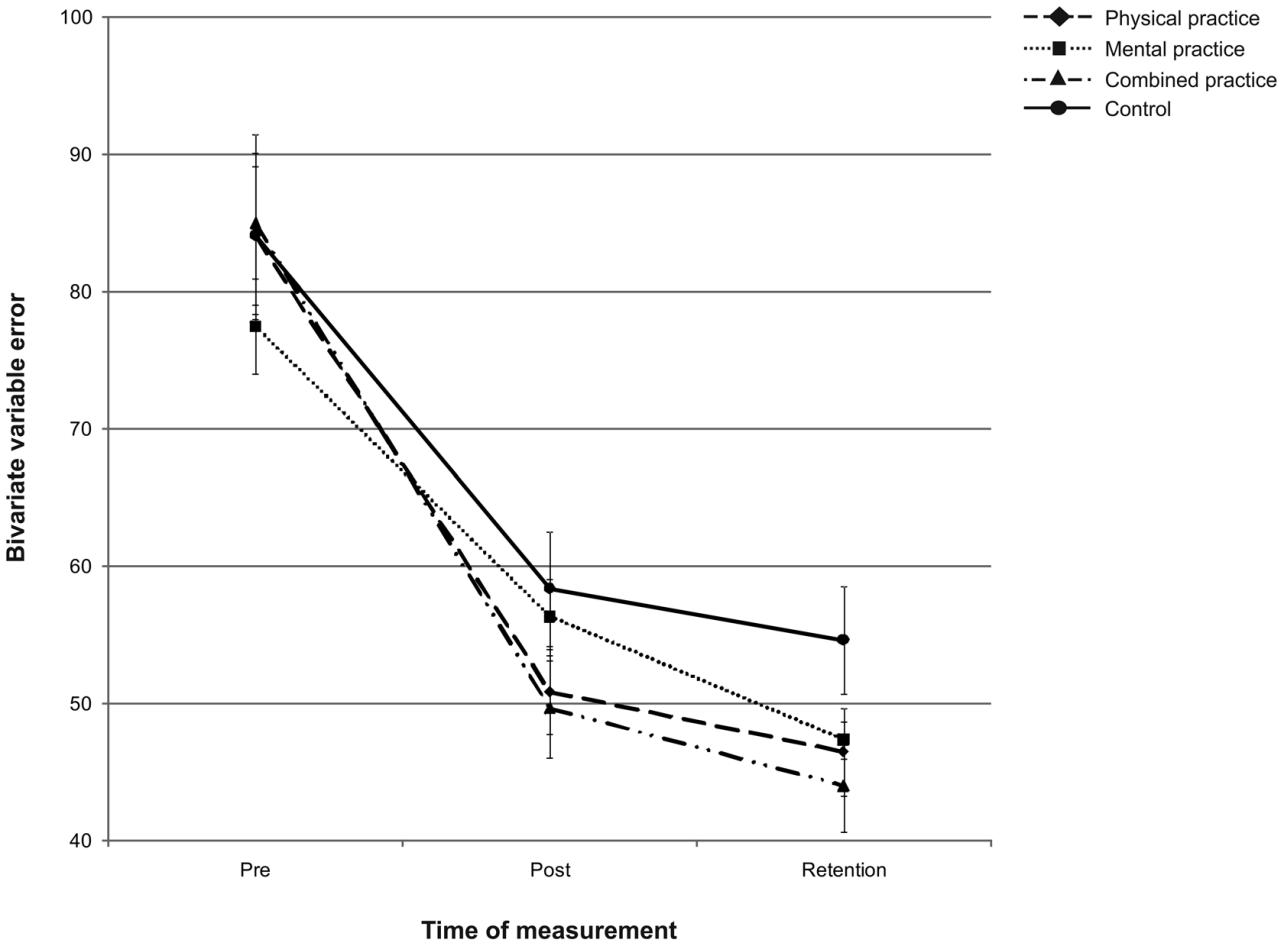
Putting consistency. Bivariate variable error (i.e., consistency) in cm from pre-test to post- and retention-test. The different lines relate to the different groups. Error bars represent standard errors.

For the four groups, putting performance from pre-, to post- and to retention-test is displayed in Figures 5 and 6. As seen in Figure 5 and 6, the physical and the mental-physical combined practice groups performed more accurately and consistently after the acquisition phase, followed by the mental practice group, whereas the control group performed worst. After a three day retention interval, however, the mental-physical combined practice group performed with the greatest accuracy and consistency followed by the physical and the mental practice groups, while the control group again performed worst (cf. Figure 5 and 6).

Regarding the acquisition phase, a one-way MANCOVA on post-test scores of MRE and BVE revealed a significant main effect of group, Wilks' Lambda = .750, *F*(6,90) = 2.326, *p* = .037, *η_p_* ^2^ = .133, 1-β = .784. Subsequent one-way ANCOVAs revealed a main effect of group for MRE, *F*(3,46) = 3.218, *p* = .031, *η_p_* ^2^ = .173, 1-β = .704 as well as for BVE, *F*(3,46) = 3.416, *p* = .025, *η_p_* ^2^ = .182, 1-β = .733. For MRE, pairwise comparisons incorporating a Holm-Bonferroni correction revealed no significant differences among the groups. For BVE, pairwise comparisons revealed that the combined practice group performed with more consistency compared to both the mental practice group (*p* = .005; α_crit_ = .008) and the control group (*p* = .009; α_crit_ = .010) post practice. The physical practice group, however, did not perform significantly different compared to either the mental practice group (*p* = .032; α_crit_ = .013), or the control group (*p* = .052; α_crit_ = .017). Regarding retention, a one-way MANCOVA on retention-test scores of MRE and BVE revealed no significant main effect of group, Wilks' Lambda = .849, *F*(6,90) = 1.279, *p* = .275, *η_p_* ^2^ = .079, 1-β = .479.

Taken together, although the groups did not show differences in learning in terms of putting accuracy, clear differences were observed in terms of putting consistency such that the combined practice led to more consistent putting compared to both mental practice only and no practice. However, these differences between groups did not persist over the three day retention interval.

## Discussion

In the present study, we investigated the effect of three different types of practice (mental practice, physical practice and their combination) in comparison to a no practice control group on both the performance and the mental representation structure of a complex movement during early skill acquisition. Overall, findings clearly denote order formation of basic action concepts of the putt together with improvements in putting performance. Interestingly, both types of practice involving imagery rehearsal (i.e., mental practice and combined practice) led to more structured and more elaborate representations, compared to physical practice and no practice.

While the mental representation structure of the control group and the physical practice group changed only marginally over time, the representation structure of the mental practice and the combined practice group elicited distinct changes over practice. Both after acquisition and after a retention interval of three days, the dendrograms of the mental practice as well as the combined practice group revealed four meaningful cluster, pertaining to functional aspects of the movement, and assignable to three movement phases in a golf putt (i.e., preparation, forward swing and impact, attenuation). Furthermore, changes in representation structures reflected a development towards a reference structure as indicated by increases in adjusted rand indices from pre-, to post-, and to retention-test. In contrast, the dendrograms of the control and the physical practice group revealed only minor changes over time. While for both groups one cluster relating to attenuation was evident after acquisition, the two dendrograms differed after a retention interval of three days. Specifically, the control groups mean dendrogram reflected two clusters pertaining to the beginning and the end of the movement (i.e., preparation and attenuation), whereas the physical practice group's dendrogram consisted of a cluster pertaining to the main phase of the movement (i.e., forward swing and impact). However, the small increases in adjusted rand indices from pre-, to post-, and to retention-test reflect only minimal development towards the reference representation. Thus, the mental and mental-physical combined practice led to more elaborate representation structures, more closely resembling an optimal representation, compared to the physical and no practice.

The results of the present study extend research on mental representations of complex action. Early research in this field, relating mental representation structure and skill level, has shown that high skill-level is associated with high order formation, and that low skill-level is associated with low order formation in long-term memory [15]. Recently, Frank et al. [23] demonstrated that practice leads to functional adaptations in one's mental representation of a complex action. Employing a similar design, the present study both replicates and extends findings reported by Frank et al. [23]. Similar to the study of Frank et al. [23], mental representation structure were found to develop over the course of practice. More importantly, however, the present study extends findings obtained by Frank et al. [23] by showing that mental practice adds to the adaptation process leading to even more elaborate mental representations compared to physical practice alone. Specifically, mental practice as well as combined mental-physical practice led to more structured representations than physical practice only and no practice. More specifically, mental representations of the putt were more similar to the reference structure for the practice groups involving mental practice of the skill than for the groups involving either physical practice only or no practice of the skill. From this, mental practice seems to lead to more developed mental representations than physical practice during early skill acquisition.

Interestingly, the mental representations of the four groups revealed slightly different patterns prior to the acquisition phase (see Figures 1a, 2a, 3a, 4a). To what extent this might influence the rate of representation development is unclear. To date, no research has examined whether the rate of development is influenced by the degree of structure in one's initial mental representation. In other words, it is conceivable that more or less structured initial representations may relate to the speed at which the structures change over the course of a practice interval. Consequently, future research is needed to clarify this point and help shed light on the learning process.

With respect to outcome performance, the combined practice led to more consistent putting performance over the course of learning compared to both mental practice only and no practice in the present study. This is in line with findings from previous research suggesting that a combination of physical and mental practice is most effective for the learning of a new motor skill [37]. While the degree to which the groups learned during skill acquisition was influenced by practice type in the present study, these differences did not persist over the course of three days of no practice. Similar to other studies investigating the effect of mental practice on the retention of a motor skill [66], the groups did not differ in their retention performance of the acquired putting skill over the course of the retention interval.

While differences in putting consistency according to practice type were obvious after acquisition phase, no differences were found in putting accuracy in the present study. That is, participants differed in how consistent their putting was, but not in how accurate each putt came to the target. Moreover, physical practice did not significantly differ from either mental or no practice, neither in terms of accuracy nor in terms of consistency. Two main reasons may have caused the lack of differences during acquisition phase. First, as reflected by the minor changes in mental representation structure, participants in the control group seem to have learned from test trials. Thus, increases in putting performance for the control group may be due to repeatedly executing the putt during test-days. Second, the lack of differences may also be due to the relative short length of the study. Specifically, too few practice sessions during acquisition phase may have resulted in the lack of clear differences between the groups. This may also be a reason for the finding that the four groups did not differ in their ability to retain their level of putting skill over three days of no practice. It is likely that larger differences would emerge over a greater length of practice. Future studies, therefore, should consider utilizing fewer trials during test days and more practice sessions during the acquisition phase to prevent this possible confound.

Whereas the groups involving physical practice (i.e., PP+CP) elicited the best putting performance after practice, those groups practicing mentally (i.e., MP+CP) revealed more elaborate representation structures after practice compared to groups who did not practice mentally. These differences pertain to distinct mechanisms underlying mental practice and physical practice. In other words, each of the groups may have learned in different ways. Learning induced by mental practice may primarily operate through and find expression on the cognitive level, whereas learning via physical practice may primarily operate through and find expression on the motor output level. In this light, it seems plausible that the two groups involving mental practice elicited more developed mental representations than the groups not practicing mentally. To explain, mental practice can be considered an “offline” process requiring primarily the re-creation of an experience from memory while covertly imagining a movement (cf. distinction between online task performance (i.e., real-time skill execution) and offline task performance (i.e., no real-time skill execution, no overt act) [67]). As there is no online information available during imagery, this process is thought to rely on memorial information only [30]. Thus, we propose that mental practice may work via the structuring of memorial information (i.e., the structuring of mental representation), and as such causes adaptation processes within the motor system. In contrast, physical practice, being an online process, requires the online integration of perceptual feedback during overt movement execution, and therefore does not primarily rely on the offline reconstruction of an experience from memory. Accordingly, physical practice applies via the integration of sensory information and as such promotes adaptation processes in this manner. Taken together, we propose that, while physical practice causes feedback-induced online adaptation, mental practice may cause memory-induced offline adaptation. In this regard, the memory-induced offline adaptation may have led to a cognitive structuring advantage in the sense of more structured memorial information on the movement (i.e., more developed mental representations of the putt) in the two groups that involved mental practice.

It seems quite interesting that, whereas mental and mental-physical combined practice led to more elaborate representation structures compared to physical and no practice, this difference was not fully expressed on the performance level in the present study. Specifically, although the findings of the present study point to the idea that mental practice in early skill acquisition may help to structure mental representation more than physical practice, this cognitive structuring advantage itself does not seem to transfer one-to-one to the motor output level. Being an “offline” process, this cognitive structuring itself seems to not immediately lead to better motor performance. It might be the case that this cognitive advantage does not turn into a performance advantage, unless online feedback is available and is being integrated. Accordingly, although the mental-physical combined practice group performed equally to physical practice in the present study, a closer look at the data points to the possibility that combined practice may be even superior to physical practice after a greater amount of practice. In fact, the combination of mental and physical practice has been suggested to be most effective in improving performance [37]. In this sense, one might speculate that the controllability of the motor system can best be achieved via both memory-induced offline adaptation (i.e., mental practice) and feedback-induced online adaptation (i.e., physical practice). Accordingly, future research might focus on long-term and transfer effects of mental and physical practice on both the performance and the representation of a motor skill.

What's more, the findings of the present study fit well into the body of research on the cognitive-motor hypothesis [68]-[71], and even extend it as we will elaborate in the following. The cognitive-motor hypothesis states that mental practice is more effective in cognitive tasks compared to motor tasks. That is, while mental practice is suggested to be effective both for cognitive and motor tasks, this hypothesis differentiates such that cognitive tasks are suggested to benefit even more from mental practice compared to motor tasks. Thus, the more cognitive a task is, the more it might benefit from mental practice. Up to now, findings largely support this hypothesis: although mental practice has been found to be effective in motor tasks [72], effect sizes reported in the meta-analysis conducted by Driskell et al. [32] were greater for cognitive tasks (*d* = .69) than for motor tasks (*d* = .34). To explain, the typical design of these studies examining the cognitive-motor hypotheses consists of two groups practicing mentally, each practicing a different task: one group practicing a cognitive task, and one group practicing a motor task. That is, two different tasks (i.e., one motor and one cognitive task) are employed in order to examine the influence of mental practice on resulting performance [69], [70]. However, to our knowledge, no study has been conducted so far that takes into account both the cognitive and the motor level within one task. Thus, no statements can be made so far whether mental practice affects more the cognitive compared to the motor level within a motor task. In the present study, we employed one task (i.e., golf putting) and examined the effect of mental practice on two different variables, one “cognitive” variable (i.e., mental representation structure) and one “motor” variable (i.e., putting performance). Thus, we used a within-task design, taking into account both the cognitive and the motor level of the golf putt. If we related the research question of the present study back to the cognitive-motor hypothesis, one would expect that mental practice would affect the cognitive structures to a larger degree than the motor output of a motor task. That is exactly what we found in the present study.

It seems important to note that oftentimes in mental practice studies, a potential lack of differences in performance according to practice type results in conclusions such that mental practice is not effective in novices. This is, of course, true with respect to performance. However, these studies do not take into account covert processes. Yet, according to learning theories, proposing that first stages of learning are primarily cognitive in nature, one might expect that changes evoked by mental practice (i.e., a cognitive type of practice) primarily take place on the cognitive level in early skill acquisition, and that these changes may not be transferred one-to-one on to the motor level without additional physical practice (i.e., a motor type of practice during which the performer repeatedly receives actual perceptual feedback). Accordingly, one would expect mental practice to especially affect the development of these cognitive processes. For the host of studies reporting no differences according to practice type, this would not necessarily mean that there were no differences between groups, but perhaps that the variables that may elicit these differences had not been measured. With the present study, we were able to show that, although not obvious from overtly observable putting performance, mental practice covertly helped to develop mental representation structure in novices.

In sum, the results of the present study clearly demonstrate that practice leads to functional adaptations in the representation structure of complex action, and that mental practice supports this adaptation, leading to even more elaborate representations. While research in the field of mental practice has largely focused on overtly observable performance effects during early skill acquisition, thereby mostly neglecting the investigation of covert cognitive effects, we showed that repeatedly imagining a movement affects the development of one's underlying mental representation structure. Building on these findings, it would be of interest to learn more about the adaptation of mental representation structure on the way to expertise. From a theoretical point of view, future research might focus on the question how different (mental) practice conditions (e.g., duration, scheduling, composition of practice) contribute to the development of mental representation structure, and, even more importantly, what conditions are most effective in contributing to the formation of an expert structure. From an applied point of view, a valuable future objective would be to examine whether practice and mental practice tailored to the one's current skill representation (i.e., individualized physical and mental practice) [73] is more effective than standard type of practice not considering one's cognitive prerequisites. To conclude, during early phases of skill acquisition, motor learning is associated with order formation of action-related knowledge in long-term memory, and this order formation seems to be promoted by mental practice.
